# Development of Autopolymerizing Resin Material with Antimicrobial Properties Using Montmorillonite and Nanoporous Silica

**DOI:** 10.3390/pharmaceutics15020544

**Published:** 2023-02-06

**Authors:** Shuhei Otsubo, Ko Nakanishi, Kakufu Fukukawa, Ryoshun Endo, Seiichiro Yoshida, Aiko Matsumoto, Kumiko Yoshihara, Tsukasa Akasaka, Akira Hasebe, Yasuhiro Yoshida, Yoshiaki Sato

**Affiliations:** 1 Department of Orthodontics, Faculty of Dental Medicine, Hokkaido University, Kita13, Nishi7, Kita-ku, Sapporo 060-8586, Japan; 2 Department of Biomaterials and Bioengineering, Faculty of Dental Medicine, Hokkaido University, Kita13, Nishi7, Kita-ku, Sapporo 060-8586, Japan; 3 Department of Oral Functional Prosthodontics, Faculty of Dental Medicine, Hokkaido University, Kita13, Nishi7, Kita-ku, Sapporo 060-8586, Japan; 4 Industrial Research Institute, Industrial Technology and Environment Research Department, Hokkaido Research Organization, Sapporo 060-0819, Japan; 5 National Institute of Advanced Industrial Science and Technology (AIST), Health and Medical Research Institute, Takamatsu 761-0395, Japan; 6 Department of Oral Molecular Microbiology, Faculty of Dental Medicine and Graduate School of Dental Medicine, Hokkaido University, Kita13, Nishi7, Kita-ku, Sapporo 060-8586, Japan

**Keywords:** orthodontic treatment, montmorillonite, nanoporous silica, cetylpyridinium chloride, Autopolymerizing Resin Material, drug release, antimicrobial

## Abstract

Although autopolymerizing resin offers numerous applications in orthodontic treatment, plaque tends to accumulate between the appliance and the mucosa, which increases the number of microorganisms present. In this study, we added cetylpyridinium chloride (CPC) loaded montmorillonite (Mont) and nanoporous silica (NPS) to autopolymerizing resin (resin-Mont, resin-NPS) and evaluated their drug release capacity, antimicrobial capacity, drug reuptake capacity, mechanical strength, and color tone for the devolvement of autopolymerizing resin with antimicrobial properties. As observed, resin-Mont and resin-NPS were capable of the sustained release of CPC for 14 d, and a higher amount of CPC was released compared to that of resin-CPC. Additionally, resin-Mont and resin-NPS could reuptake CPC. Moreover, the antimicrobial studies demonstrated that resin-Mont and resin-NPS could release effective amounts of CPC against *Streptococcus mutans* for 14 d and 7 d after reuptake, respectively. Compared to resin-CPC, resin-Mont exhibited a higher sustained release of CPC in all periods, both in the initial sustained release and after reuptake. However, the mechanical strength decreased with the addition of Mont and NPS, with a 36% reduction observed in flexural strength for resin-Mont and 25% for resin-NPS. The application of these results to the resin portion of the orthodontic appliances can prevent bacterial growth on the surface, as well as on the interior, of the appliances and mitigate the inflammation of the mucosa.

## 1. Introduction

Autopolymerizing resin offers numerous applications in orthodontic treatment and functional orthodontic appliances such as activators, inclined bite plates, expansion plates, other orthodontic plates, Nance lingual arches used for correction, and retainers used for retention treatment. Most of these orthodontic appliances are used long-term over several years. As reported in the case of fixed appliances, plaque tends to accumulate between the appliance and the mucosa, which increases the number of microorganisms present [1,2]. In addition, bacteria can invade and contaminate the resins during long-term usage, thereby deteriorating the appliance [3]. Moreover, several studies have reported contamination in the treatment room caused by the scattering of bacteria-contaminated resin shards during grinding adjustments [4,5]. Accordingly, scholars have attempted to resolve this issue by inducing antimicrobial properties to the resins [6], for example, by directly adding chlorhexidine. Although the elution of the drug into the surrounding area temporarily exerts antimicrobial value, the duration of the antimicrobial efficacy remains an issue [7,8]. Therefore, recent studies have attempted to add various nanomaterials, such as zinc oxide [9], titanium dioxide [10], and silver particles [11], to dental materials to provide antimicrobial properties. In this study, we focused on montmorillonite (Mont) and nanoporous silica (NPS), which have nanostructures and include drug loading and reuptake capabilities.

Mont is contained in a mineral called bentonite, which is used in food products and is highly safe for biological health. Additionally, this is an inexpensive and easy-to-apply material, because it can be obtained in large quantities throughout many countries. The structure is constituted of a layer comprising a series of tetrahedra of silicate and a layer comprising a series of octahedra of aluminum hydroxide and magnesium hydroxide, with one octahedral layer located between two tetrahedral layers [12]. The partial isomorphic substitution of aluminum for magnesium in the octahedral structure creates a deficit of charge on the layer surface, thereby yielding a negatively charged layer. To improve this charge imbalance, positively charged ions such as Na^+^, Ca^2+^, K^+^, and Mg^2+^ have been incorporated between the layers. The cations between these layers are exchangeable with other cations (cation exchange capacity) [13,14] and can be utilized to incorporate drugs into the layers [14]. Matsuo et al. developed a dental adhesive material with antimicrobial properties by incorporating the cationic antimicrobial agent cetylpyridinium chloride (CPC) into Mont [14].

Moreover, NPS is a nanosized porous material, primarily composed of silicon dioxide, and its features, such as surface treatment, pore morphology, and size, can be adjusted. Thus, in recent years, several studies have applied it to drug delivery systems [15,16,17]. The negatively charged surface of NPS [18] can attract cationic antimicrobial agents and capture them in the pores. As reported, fenbufen [19], silver [20], and chlorhexidine [21,22] can be loaded for sustained release.

This study used Mont and NPS as drug carriers to develop antimicrobial autopolymerizing resins, and cationic antimicrobial agents were added to prepare autopolymerizing resins containing Mont (resin-Mont) and NPS (resin-NPS). Thereafter, drug sustained-release capacity, reuptake capacity, antimicrobial efficacy, mechanical properties, and color tone were evaluated. Moreover, CPC was used as the cationic antimicrobial agent. CPC is a quaternary ammonium salt cationic antimicrobial agent used in mouthwashes and toothpastes for application in the oral cavity, and it is effective against oral bacteria such as *Streptococcus mutans* and fungi such as *Candida albicans* [23].

## 2. Materials and Methods

### 2.1. Materials

**Figure 1:**
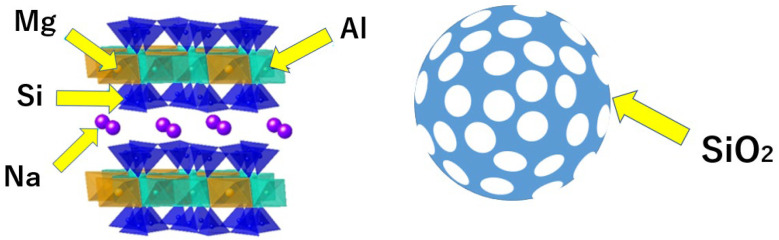
Structure of montmorillonite and nanoporous silica.

Kunipia-F (Kunimine Industries, Tokyo, Japan) was used for Mont, and the NPS (Sigma-Aldrich, St. Louis, MI, USA) exhibited a particle size of 2.0 μm with a pore size of 4.0 nm. (Figure 1) Orthocrystal (JM Ortho, Tokyo, Japan) was used as the autopolymerizing resin (Table 1), and CPC (Tokyo Chemical Industry, Tokyo, Japan) was used as the cationic antimicrobial agent.

### 2.2. Specimen Preparation

Mont powder and NPS powder were added to an aqueous solution of 10.0 wt% CPC and stirred at 50 °C and 1500 rpm for 12 h before being filtered and dried. The resulting powders were mixed with the powder component of the autopolymerizing resin at a concentration of 10.0 wt% for both resin-Mont and resin-NPS. Thereafter, the adjusted powder and liquid were mixed and cured at the mixing ratio (P/L = 1.00/0.45) described in the manufacturer’s instructions for preparing the test specimens. In particular, disc-shaped specimens (height: 1.0 mm; diameter: 10.0 mm) were used for evaluating the sustained release capacity, reuptake capacity, antimicrobial efficacy, and coloration of the antimicrobial agent, whereas rectangular specimens (width: 40.0 mm; depth: 10.0 mm; height: 1.0 mm) were used to characterize the mechanical properties. In this study, the cation exchange capacity of Mont was 1.19 mmol/g [13], and the amount of CPC that could be loaded by Mont in the specimen at a 10 wt% was calculated as 2.7 wt% of the specimen. Therefore, CPC was directly added to the autopolymerizing resin at 2.7 wt%, and its specimens were prepared following the above-stated procedure for use as a control (resin-CPC).

### 2.3. Evaluation of Antimicrobial Agent Sustained Release Capacity

The three specimens of the prepared resin-Mont, resin-NPS, and resin-CPC were immersed in 3.0 mL of distilled water at 37.0 °C for 24 h. Subsequently, the specimens were removed from the solution and immersed again in 3.0 mL of fresh distilled water. This cycle was continued for 14 d. After 14 d, the concentration of CPC obtained on each day from the supernatant solution was analyzed using a high performance liquid chromatography (HPLC) system (Nexera X2, Shimadzu Corporation, Japan) equipped with a photodiode array detector (SPD-M20A, Shimadzu Corporation, Japan). The analysis conditions are as follows [1]: column, Shim-pack Arata C18 (75 mm × 3.0 mm I.D., 2.2 μm, Shimadzu GLC Ltd., Japan); flow rate, 1.0 mL/min; mobile phase, (A) 0.1 vol% formic acid in water and (B) 0.1 vol% formic acid in acetonitrile; time program, 40% (B) (0 min)—50% (B) (4.0–4.5 min)—40% (B) (4.5–7.0 min); column temperature, 40 °C; injection volume, 50 μL; and detection wavelength, 254 nm.

### 2.4. Evaluation of Reuptake Capacity

To evaluate the CPC reuptake capacity, the three specimens were immersed in a 10.0 wt% CPC solution for 24 h at 37.0 °C for reuptake after 14 d of sustained release. After rinsing and drying, the specimens were immersed in 3.0 mL of distilled water for 24 h at 37 °C. Thereafter, the specimens were removed and immersed in 3.0 mL of fresh distilled water. This cycle was repeated continuously for 7 d. The absorption spectra of the supernatant solution obtained after 7 d were measured using liquid chromatography (Nexera X2, Shimadzu Corporation, Kyoto, Japan) to evaluate the sustained release of CPC.

### 2.5. Evaluation of Antimicrobial Efficacy

*Streptococcus mutans* (*S. mutans*, ATCC55677) was cultured in Brain Heart Infusion (BHI) (Becton Dickson and Company, Franklin Lakes, NJ, USA) medium for 24 h at 37 °C. After 24 h, the bacterial solution was measured using a spectrophotometer (Double Beam Spectrophotometer U-2910, HITACHI, Tokyo, Japan) and diluted to an optical density (turbidity) of 1.0 to prepare the bacterial solution. The resulting *S. mutans* bacterial solution was further diluted 10-fold for each experiment. The test specimens were used for antimicrobial testing 7 d, 14 d, and 7 d after reuptake and the sustained release of CPC in distilled water. One platinum loop of the adjusted *S. mutans* solution and one of each specimen were added to 5.0 mL of BHI liquid medium for each test and incubated for 24 h at 37 °C in a static condition. A control was prepared by simply adding one platinum loop of the adjusted *S. mutans* bacterial solution to the BHI liquid medium without specimens. The turbidity of the culture medium after 24 h was measured using a spectrophotometer (Double Beam Spectrophotometer U-2910, HITACHI, Tokyo, Japan) at a wavelength of 600 nm to evaluate the antimicrobial efficacy.

### 2.6. Evaluation of Mechanical Strength

The mechanical strength of resin-Mont and resin-NPS was evaluated by performing a three-point bending test. Thereafter, the measurements were recorded on a universal testing system (Model 3366, Instron, Canton, OH, USA) at a crosshead speed of 5.0 mm/min and a span of 20 mm until the specimen broke, and the flexural strength was calculated from the load at the maximum load point. In total, five identical samples were used for each test, and the autopolymerizing resin (resin-cont) was singularly used as a control.

### 2.7. Evaluation of Color Tone

The color tone of resin-Mont and resin-NPS was evaluated using a CR-20 (Konica Minolta, Tokyo, Japan) device against a black background. The three specimens were measured 7 d, 14 d, and 7 d after reuptake, respectively. A specimen of just-cured resin was prepared as a control. The L*a*b* color space was used, wherein the color difference is calculated using the following formula [24].

(1) $$\Delta {E^{*}}_{\mathrm{ab}}=\sqrt{{\left(L_{2}^{*}-L_{1}^{*}\right)}^{2}+{\left(a_{2}^{*}-a_{1}^{*}\right)}^{2}+{\left(b_{2}^{*}-b_{1}^{*}\right)}^{2}}$$

## 3. Results

### 3.1. Evaluation of CPC Sustained Release Capacity of Resin-Mont and Resin-NPS

**Figure 2:**
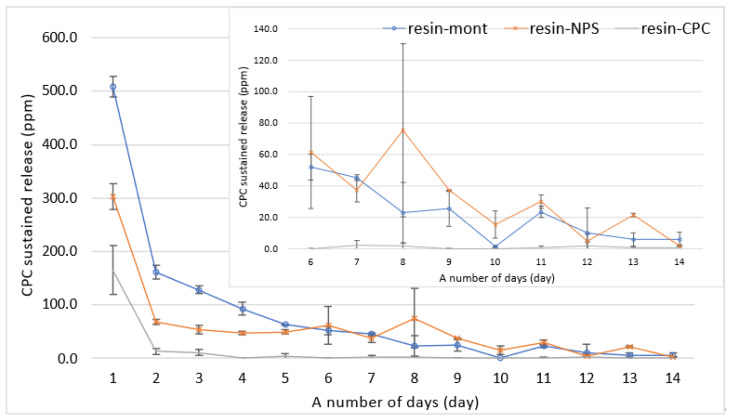
Amount of released CPC from resin-Mont, resin-NPS, and resin-CPC. The inside graph is zoomed in on days 6 through 14.

The results of the liquid chromatographic measurements of the supernatant solution are depicted in Figure 1. As observed, the sustained release of CPC displayed a gradually decreasing trend in all samples. A comparison of the sustained release indicated that the sustained release of the resin-Mont and resin-NPS samples was higher than that of resin-CPC on all days; on day 9, that of resin-CPC was below 1 ppm (under the detection limit). The amount of sustained release was higher for resin-Mont than resin-NPS on days 1–5, but after day 6, the amount of sustained-release was almost equal (Figure 2).

### 3.2. Evaluation of Reuptake Capacity

**Figure 3:**
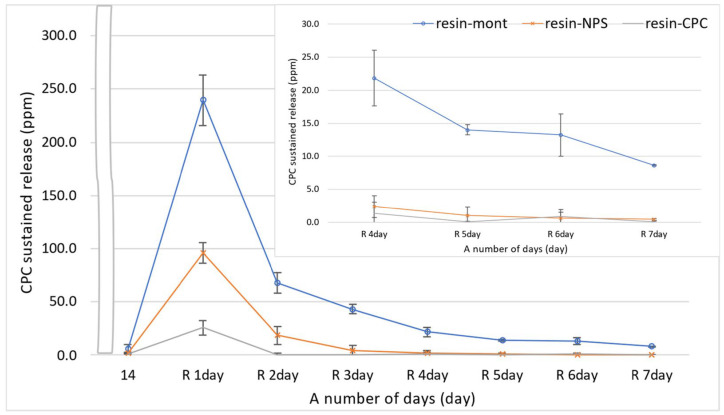
Amount of released CPC from resin-Mont, resin-NPS, and resin-CPC after reuptake. The inside graph is zoomed in on day R 4 through day R 14.

In all tests of resin-Mont, resin-NPS, and resin-CPC, the sustained release on day 1 after reuptake increased beyond that on day 14 before reuptake. Additionally, the amount of CPC sustained release diminished gradually in the same manner as before reuptake. Overall, the CPC sustained release was higher in the order of resin-Mont, resin-NPS, and resin-CPC. In particular, the sustained release of resin-Mont was higher on day 7 after reuptake than on day 14 before reuptake. However, the sustained release of resin-NPS decreased to 1.0 ppm on day 5 after reuptake and was below the sustained release on day 14 before reuptake. The sustained release of resin-CPC was below 1.0 ppm on day 2, and subsequently, decreased to the same level as on day 14 before reuptake (Figure 3).

### 3.3. Evaluation of Antimicrobial Efficacy through Antimicrobial Testing

In tests using specimens cultured for 7 and 14 d, the turbidity of all specimens was lower than that of the control, indicating antimicrobial efficacy. More specifically, the samples of resin-Mont, resin-NPS, and resin-CPC exhibited a decreasing order of turbidity and an increasing order of antimicrobial efficacy, respectively. More importantly, the higher turbidity on day 14 than on day 7 confirmed a gradual weakening of the antimicrobial efficacy.

**Figure 4:**
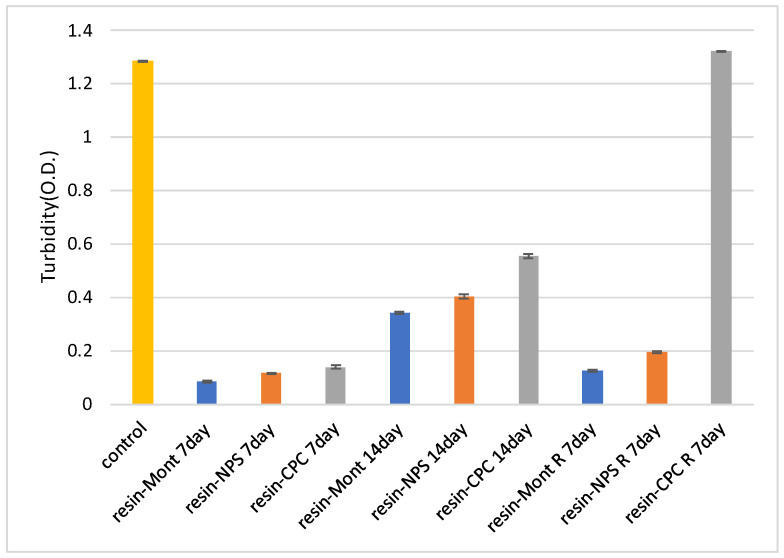
Comparison of variations in turbidity by antimicrobial test (“after reuptake” is denoted by “R”).

At 7 d after reuptake, the turbidity of resin-Mont and resin-NPS decreased in comparison to that of the control, thereby indicating antimicrobial efficacy. The turbidity of resin-Mont was less than that of resin-NPS, thereby indicating the higher antimicrobial efficacy of resin-Mont. The turbidity of both resin-Mont and resin-NPS was less than the results at 14 d before reuptake, implying that the reuptake of CPC enhanced the antimicrobial efficacy. In contrast, the resin-CPC exhibited high turbidity compared to the control and exhibited no effect under reuptake (Figure 4).

### 3.4. Evaluation of Mechanical Strength

**Figure 5:**
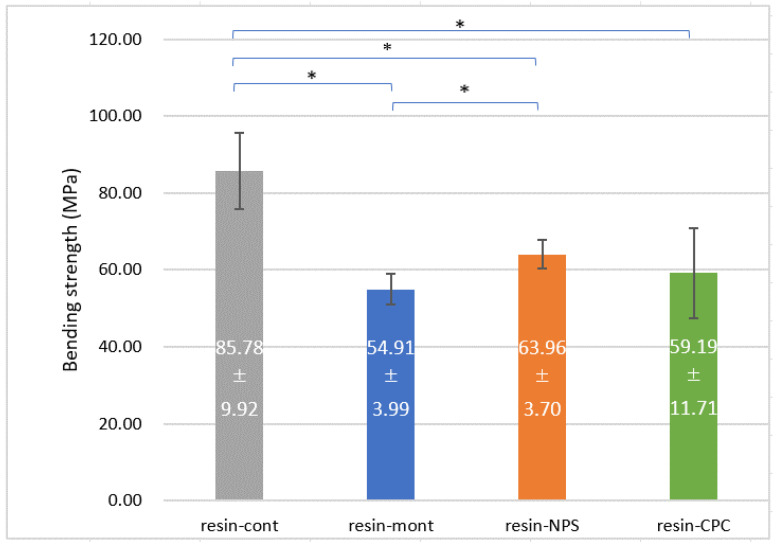
Results of 3-point bending test (*: *p* < 0.05).

The flexural strength was higher in the order of resin-cont (85.8 MPa), resin-NPS (64.0 MPa), resin-Mont (54.9 MPa), and resin-CPC (59.2 MPa), and the mechanical strength diminished with the addition of Mont, NPS and CPC (Figure 5).

Compared to resin-cont, resin-NPS, resin-Mont, and resin-CPC exhibited a 25%, 36%, and 31% reduction in bending strength, respectively.

### 3.5. Evaluation of Color Tone

**Figure 6:**
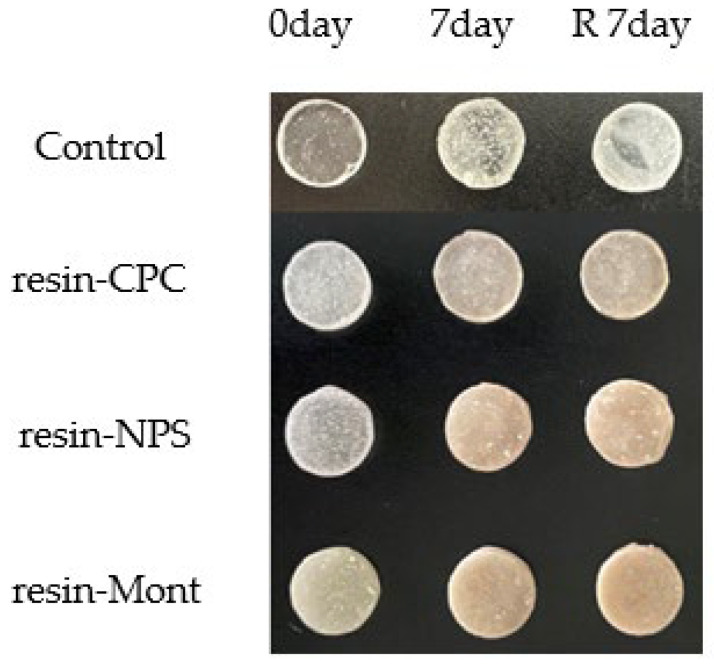
Change in color tone (“after reuptake” is denoted by “R”).

Among all the specimens, the control exhibited the least value of L*, and a* and b* were almost zero. A comparison of the specimens on day 0 indicated a higher value of L* in the order of resin-Mont, resin-NPS, resin-CPC, and the control. Compared to the control, all specimens exhibited a higher b* and a marginally lower a*. At day 7, L* decreased and darkened for all specimens, and a* and b* were higher. After reuptake, L* of resin-Mont and resin-NPS increased and brightened compared to that on day 7, whereas L* further reduced and darkened in comparison to that on day 7 for resin-CPC. Overall, no variations existed in a* and b* for resin-CPC, both a* and b* were higher for resin-Mont, and a* was smaller for resin-NPS. Furthermore, if a color difference of 2.3 was evaluated as a just-noticeable difference [24], the color difference of the specimens on day 0, day 7, and day 7 after reuptake exceeded the just-noticeable difference in comparison to the control for all specimens. Moreover, the color tone varied according to the recognizable extent. This value was higher in the order resin-Mont, resin-NPS, and resin-CPC (Figure 6 and Table 2).

## 4. Discussion

The present findings indicate that the addition of Mont or NPS to autopolymerizing resin enables the sustained release of CPC over a long duration, as well as its reuptake. Compared to resin-CPC, resin-Mont exhibited a higher sustained release of CPC in all periods, both in the initial sustained release and after reuptake. Theoretically, the same amount of CPC was added to the specimens, suggesting that using Mont as a drug carrier would enable the sustained release of CPC. Namba et al. [25] added CPC directly to light-cured bonding materials and reported almost no sustained release. In contrast, Matsuo et al. added CPC directly to self-etching adhesive in a one-step method and confirmed the sustained-release of CPC for several days. The experimental results in this study were similar to those of Matsuo et al. [13]. Although the particulate CPC in resin-CPC was encapsulated in autopolymerizing resin by polymerization, the CPC particles were not bonded to the resin to ensure a certain mobility and gradual release [25]. However, owing to the high compactness of the resin interior, only the surface or extremely near-surface CPC can be released, whereas the release of the CPC encapsulated in deeper layers fails. Moreover, the CPC in resin-Mont was loaded between the layers of Mont and gradually released. Therefore, CPC can be released if a portion of the Mont (interlayer exit) is present on the surface or within the surface layer. In addition, it is possible that CPC was released from the deeply encapsulated Mont in the resin through the other interlayer of Mont. Therefore, the amount of sustained-release presumably increased upon using Mont as a carrier instead of using only CPC with the resin. In resin-NPS, on the other hand, the negatively charged surface of the NPS [18] electrically attracts, loads, and releases CPC in a sustained manner. Therefore, resin-NPS exhibits a sustained release of CPC from the NPS present on the surface or within the surface layer. Furthermore, sustained release may occur from the deeply encapsulated NPS in the resin through the other NPS, similar to the case of Mont. Until day 5, the sustained release of CPC was greater with resin-Mont than with resin-NPS, and from day 6 onward, the sustained release was almost equal between resin-Mont and resin-NPS. This may be caused by the fact that the electrical force with Mont loading the drugs was weaker than that with NPS, facilitating sustained release, and because the amount of CPC that Mont can load was larger than that of NPS for the same mass. In this study, resin-Mont and resin-NPS displayed a sustained release of CPC for several days after reuptake, whereas resin-CPC exhibited sustained release on only day 1 after reuptake. This is because only the CPC attached to the resin surface was released, and the reuptake into the autopolymerizing resin was not possible without employing a carrier. Moreover, resin-Mont delivered a higher sustained release after reuptake compared to resin-NPS, which could be attributed to the differences in the amount that can be taken in. Nonetheless, further investigation is required to understand this phenomenon in detail.

The results of the antimicrobial test displayed that resin-Mont, resin-NPS, and resin-CPC maintained antimicrobial efficacy for 14 d, with resin-Mont and resin-NPS maintaining antimicrobial efficacy on day 7 after reuptake. As observed, the addition of a drug carrier enables the reuptake of CPC and the maintenance of antimicrobial efficacy for a long duration. Considering the relationship with CPC sustained release, resin-NPS displayed higher antimicrobial efficacy on day 7 after reuptake than on day 14 before reuptake, although the amount of releasing CPC was less on day 7 after reuptake than on day 14 before reuptake and was below the traceable concentration. In addition to the incorporation of CPC into NPS during reuptake, the amount of CPC electrically adhered to the NPS surface increased. It was not released in a sustained manner, but the material surface demonstrated antimicrobial efficacy. In this regard, Namba et al. [25] reported that CPC, which is affixed to the bonding material and does not undergo sustained release, exhibited antimicrobial efficacy. In addition, CPC was loaded onto NPS and mixed with resin to prepare the specimen, which may have weakened the electrical adhesion between the NPS and CPC owing to the influence of methyl methacrylate, tertiary amines, and other factors. As the organic components do not exert any influence during reuptake, the NPS and CPC exposed on the surface were firmly attached via electrical charges. Regardless, further studies are required to clarify the phenomena such as the mechanism of electrical attachment and sustained release.

In this study, *Streptococcus mutans* was used to evaluate antimicrobial efficacy. As *Streptococcus mutans* is a well-known cause of dental caries [26], we believe that the use of this development can be expected to prevent dental caries from occurring around appliances. With prolonged use of resin-based appliances, plaque deposits containing large amounts of *Candida albicans* are observed on the mucosa under the resin, as well as on the resin itself [27]. Nikawa et al. reported that the adhesion capacity of *Candida albicans* on the resin and the underlying mucosa is related to hydrophobic and electrostatic interactions [28]. However, Khalid et al., in their report on the symbiosis of *Candida albicans* and *Streptococcus mutans*, posited that this factor cannot sufficiently prove a strong attachment [29]. In addition, Howard et al. reported that *Candida albicans* adheres firmly to resin by aggregation with oral microorganisms such as *Streptococcus mutans* [30]. Conclusively, the antimicrobial test results obtained with the application of Mont or NPS as a drug carrier in this study demonstrated effectiveness against *Candida albicans* adhesion to the resin and underlying mucosa due to antimicrobial efficacy against *Streptococcus mutans*, and they are expected to be effective against inflammation of the mucosa. In addition, CPC delivered direct fungicidal efficacy on *Candida albicans*. In the future, the antimicrobial efficacy against *Candida albicans* should be further verified.

The mechanical strength decreased in the order of resin-Mont, resin-NPS, and resin-cont, wherein the specimen containing Mont exhibited the lowest mechanical strength. The inorganic substance Mont can be fabricated as a pseudo-organic material using its cation exchange capacity to exchange the interlayer cations with organic cations, as it manifests affinity for organic materials such as autopolymerizing resins [31]. CPC bears a long carbon chain, C21H38NCl and includes a large organic component [32]. Therefore, we predicted that loading CPC into the interlayer of Mont can render Mont pseudo-organic, thereby improving its affinity with the resin, and mechanical properties of the resin including Mont loading CPC will not vary significantly. However, in the present study, the addition of 10 wt% of Mont to the autopolymerizing resin deteriorated the mechanical strength compared with the resin-cont, and marginally less than that of resin-NPS. Mousavi et al. [33] added Mont to silicon acrylate and evaluated its mechanical strength. The bending strength displayed a maximum value at an addition of 0.03 wt%, whereas the mechanical strength deteriorated with further additions. In this regard, the mechanical properties can be improved up to a certain concentration owing to the improved dispersion of Mont in the matrix and the increased interfacial adhesion area between the substrate and Mont. However, beyond a certain concentration, the mechanical properties deteriorate because of the tendency of Mont to form aggregates [32]. In the present study, the addition of 10 wt% caused the internal agglomeration of Mont, which may have reduced the mechanical strength. Thus, further research should quantitatively determine the optimum amount of Mont that should be added to deliver adequate antimicrobial efficacy without degrading the mechanical strength. Regarding the mechanical strength of resin-NPS, Zhang et al. added chlorhexidine-loaded NPS to a light-cured resin and immersed it in water. They reported that the bending strength decreased with an increasing amount of added NPS compared to raw resin, which was related to the dispersion of the filler particles [22]. Herein, the deterioration in the mechanical strength owing to the addition of NPS to the autopolymerizing resin is expected as a result of the aggregation of NPS at an addition of 10 wt%. Nakamura et al. reported that the addition of 10 wt% NPS to glass ionomer cement did not alter the compressive strength, but increasing the amount of added NPS can inhibit the curing reaction of glass ionomer cement, thereby significantly decreasing the compressive strength [34], although this is different from the polymerization reaction of resin. Possibly, the excess amount added in this study inhibited the polymerization reaction of the resin. Endo et al. [35] reported that the number of cations to be loaded can be adjusted by varying the pore size. Upon selecting the optimal pore size for loading CPC, the amount of added Mont may be reduced, while maintaining the same amount of sustained release of CPC. In addition, the size of the filler particles affects the mechanical strength of the composite [36,37,38]. Therefore, the addition amount can be potentially increased without decreasing the mechanical properties by selecting the optimum particle size. In the future, the optimization of the particle size and pore size is necessary to provide a sustained release of CPC in sufficient amounts, while maintaining mechanical strength.

Regarding the color tone, the color difference in the autopolymerizing resin exceeded the just-noticeable difference from resin-cont at the time of adding Mont, NPS, and CPC (day 0). The color tone varied at day 0, day 7, and day 7 after reuptake for all specimens, indicating the temporal variations in the color tone. The color tone became more reddish and yellowish with the addition of only CPC, but not Mont or NPS, indicating that the inclusion of CPC may alter the color tone of the resin over time. In the case of using NPS or Mont as a carrier, the color tone varied more with the addition of Mont. In a report by Yamagata et al., organically treated Mont was added to PMMA, and the total light transmittance and haze were evaluated. As the addition rate of the organically treated Mont increased, the transparency became more brownish, wherein the total light transmittance decreased to 78.3% and the haze increased significantly for specimens with a 10% addition [39]. In this study, the color tone altered to brown, probably because of the original color of the Mont itself. In contrast, Jiangkongkho et al. added silanized NPS to PMMA, evaluated the color tone, and determined that the color tone may be affected by an increasing addition amount [40]. Accordingly, the present findings attested to similar variations in the color tone.

**Figure 7:**
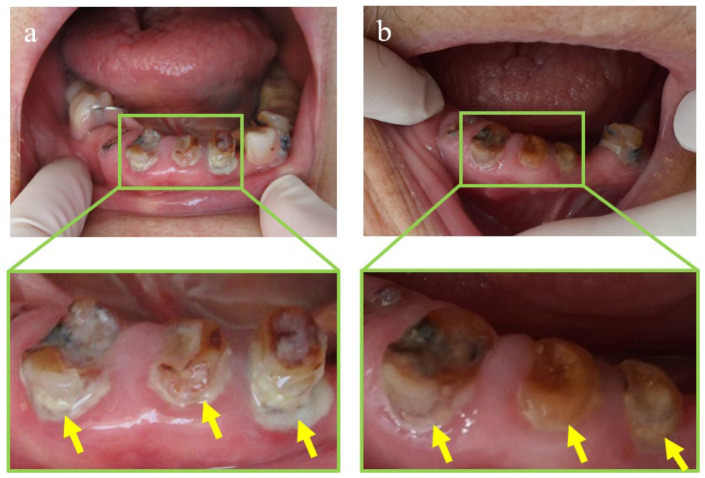
Effect of tissue conditioner CPC: (**a**) before use of tissue conditioner CPC; (**b**) after 1 week of use. Tissue conditioner CPC decreased the plaque around the tooth stump.

From a clinical perspective, we believe that the application of these results to producing the resin portion of the orthodontic appliances can prevent bacterial growth on the surface, as well as on the interior, of the appliances and improve the inflammation of the mucosa caused by using such appliances with contaminated resin. In orthodontic treatments, the same appliance is often used continuously over a period of several years. Therefore, even with adequate cleaning and management, bad odors caused by tartar deposition and bacterial growth on the apparatus cannot be avoided. Specifically, cleaning fixed appliances is challenging because patients cannot remove the appliance themselves, and even if the appliance is removed and thoroughly cleaned during the patient’s visit to the dental clinic, the level of cleaning is still insufficient. The development of this study is extremely effective for maintaining the hygiene of such orthodontic appliances. During the dynamic orthodontic treatment period, patients are often asked to visit the clinic every month for adjusting the appliance. The reuptake of the drug at the time of visit can maintain antimicrobial efficacy throughout the duration of the orthodontic treatment. In addition, although the patient visits the clinic every few months during the retention treatment period, a removable appliance is often used for treatment. A removable appliance enables the patient to maintain antimicrobial efficacy via reuptake of the drug every two to four weeks by themselves. The reuptake of the drug in this development is a simple procedure of immersion in a solution, which can be easily performed by patients on their own. In addition, CPC is released gradually on the surface as well as the interior of the appliance including the surrounding area. Currently, the only dental material on the market that releases drugs on the surface and surrounding area is a short-term resilient lining material for removable dentures, called tissue conditioner CPC. This material contains Mont loading CPC, which gradually releases CPC for a few weeks. In clinical practice, when tissue conditioner CPC was used on elderly patients with poor oral hygiene due to inadequate oral cleaning, it showed great efficacy in that the plaque around the tooth stump decreased due to the release of CPC. (Figure 7) As for tissue conditioner CPC, the developed application in this study can be also be expected to produce certain improvement in the oral hygiene field.

## 5. Conclusions

This study prepared resin-Mont and resin-NPS using CPC-loaded Mont and NPS and evaluated the variations in their drug release capacity, antimicrobial capacity, drug reuptake capacity, mechanical strength, and color tone. In particular, resin-Mont and resin-NPS were capable of the sustained release of CPC for 14 d, and a higher amount of CPC was released compared to that of resin-CPC. In addition, resin-Mont and resin-NPS were able to reuptake CPC. Moreover, the antimicrobial studies demonstrated that resin-Mont and resin-NPS could release effective amounts of CPC against *Streptococcus mutans* for 14 d and 7 d after reuptake. However, the mechanical strength decreased with the addition of Mont and NPS, with a 36% reduction observed in flexural strength for resin-Mont and 25% for resin-NPS. In conclusion, Mont and NPS are useful for the development of new antimicrobial dental base materials. However, in the future, the addition amount of Mont and NPS to autopolymerizing resin should be verified to ensure the maintenance of its mechanical strength.

## Figures and Tables

**Table 1:** Components of Orthocrystal.

Component	Contents
Powder	copolymer of methacrylic ester
	other
Liquid	methyl methacrylate
	tertiary amine
	coloring agent
	other

**Table 2:** Change in color tone test results (“after reuptake” is denoted by “R”).

Sample	Day	L*	a*	b*	Color Difference
control	0 day	30.6	0.02	−0.04	0
	7 day	33.1	0.06	0.6	2.58
	R 7 day	34.6	−0.1	0.73	4.08
resin-CPC	0 day	41.7	−0.23	0.77	11.3
	7 day	39.13	0.77	2.97	9.08
	R 7 day	34.9	0.77	2.7	5.15
resin-Mont	0 day	56.5	−0.87	7.77	27.07
	7 day	54.47	3.13	10.83	26.41
	R 7 day	58.33	3.63	12.17	30.52
resin-NPS	0 day	45.43	−0.2	1.37	14.9
	7 day	42.07	3.27	6.87	13.77
	R 7 day	50.27	2.7	6.97	21.05

## Data Availability

The data presented in this study are available from the corresponding author, K.N., upon reasonable request.
